# Cross-cultural adaptation of the CHO-KLAT for boys with hemophilia in rural and urban china

**DOI:** 10.1186/1477-7525-10-112

**Published:** 2012-09-15

**Authors:** Runhui Wu, Jishui Zhang, Koon Hung Luke, Xinyi Wu, Tricia Burke, Ling Tang, Man-Chiu Poon, Xiaojing Li, Min Zhou, Jing Sun, Marrisa Hang, Victor Blanchette, Nancy L Young

**Affiliations:** 1 Department of Haematology, Beijing Children’s Hospital and Capital Medical University, Beijing, China; 2 Department of Neurology, Beijing Children’s Hospital and Capital Medical University, Beijing, China; 3 Division of Haematology, Children’s Hospital of Eastern Ontario, Ottawa, ON, Canada; 4 School of Rural and Northern Health, Laurentian University, 935 Ramsey Lake Road, Sudbury, Ontario, Canada; 5 Departments of Medicine, Pediatrics and Oncology, University of Calgary, Foothills Hospital, Calgary, Alberta, Canada; 6 Department of Haematology, Chengdu Women and Children’s Central Hospital, Chengdu, Sichuan Province, China; 7 Department of Haematology, Nanfang Hospital, Guangzhou, Guangdong Province, China; 8 Division of Hematology and Oncology, Hospital for Sick Children, Toronto, and the Department of Pediatrics, University of Toronto, Toronto, Ontario, Canada

**Keywords:** Quality of life, Hemophilia, China, Children, Cross-cultural studies

## Abstract

**Background:**

Quality of life (QoL) is increasingly recognized as an important outcome measure in clinical trials. The Canadian Hemophilia Outcomes-Kids Life Assessment Tool (CHO-KLAT) shows promise for use in China.

**Objective:**

To adapt the CHO-KLAT version 2.0 for use in clinical trials in China.

**Methods:**

Forward and back translations of the CHO-KLAT_2.0_ were completed in 2008. Between October 2009 and June 2010, a series of 3 focus groups were held with 20 boys and 31 parents in rural and urban China to elicit additional concepts, important to their QoL, for the Chinese CHO-KLAT_2.0_. All of the items identified by boys and parents were reviewed by a group of experts, resulting in a Chinese version of the CHO-KLAT_2.0_. This version underwent a detailed cognitive debriefing process between October 2010 and June 2011. Thirteen patient-parent pairs participated in this cognitive debriefing process until a stable and clearly understood Chinese version of the CHO-KLAT_2.0_ was obtained.

**Results:**

The initial back translation of the Chinese CHO-KLAT_2.0_ was slightly discrepant from the original English version on 12 items. These were all successfully adjudicated. The focus groups identified 9 new items that formed an add-on Socio-Economic Context (SEC) module for China. Linguistic improvements were made after the 2^nd^, 5^th^, 7^th^ and 13^th^ cognitive debriefings pairs and affected a total of 18 items. The result was a 35 item CHO-KLAT_2.0_ and a SEC module in Simplified Chinese, both of which have good content validity.

**Conclusion:**

This detailed process proved to be extremely valuable in ensuring the items were accurately interpreted by Chinese boys with hemophilia ages ≤18 years. The need for the additional SEC module highlighted the different context that currently exists in China with regard to hemophilia care as compared to many Western countries, and will be important in tracking progress within both rural and urban China over time. Changes based on the cognitive debriefings suggest that expert verbatim translation alone is not sufficient. The Chinese version of the CHO-KLAT_2.0_ is well understood by boys with hemophilia in China. Next steps will be to test its construct validity and reliability in boys with hemophilia in China.

## Background

Hemophilia is a congenital, X-linked recessive bleeding disorder resulting in low levels of either factor VIII (hemophilia A) or factor IX (hemophilia B) that affects approximately 1 in 5,000 (hemophilia A) and 1 in 20,000-30,000 (hemophilia B) live male births. [1] Spontaneous recurrent bleeding into the joints, muscles and soft tissues often results in impairments in function. This is most common in cases with severe hemophilia, defined by a circulating factor VIII or IX level of < 1%. These impairments may lead to diminished quality of life (QoL). Thus, QoL is an important outcome for clinical research in hemophilia.

Over the last 2 decades there has been significant improvement in hemophilia care in China promoted by the World Federation of Hemophilia (WFH), beginning in 1993. The development of WFH twinning programs with leading Canadian hemophilia treatment centers began in 1997, followed by the development of a 6-hospital collaborative network in China (Tianjin, Guangzhou, Shanghai, Beijing, Hefei, and Jinan). [2] In the past there has been limited availability of clotting factor concentrates in China. Recent literature indicates that 90% of boys in China over the age of 6 years have significant joint arthropathy, [3] which may reflect a gap in access to treatment. China is now benefiting from increased access to factor replacement therapy. In addition, hemophilia treatment centers are beginning to be developed in many cities. These are based on the comprehensive hemophilia care model. The recent changes in management of hemophilia in China present a unique opportunity to study the impact of factor replacement therapy and enhanced hemophilia care on the quality of life of boys with hemophilia.

The Canadian Hemophilia Outcomes-Kids Life Assessment Tool (CHO-KLAT) [4,5] was developed in Canada to measure the quality of life of boys with hemophilia. Because of its strong measurement properties [5] and recent cross-cultural validation in many European countries, it was selected for cross-cultural adaptation for use in China. Disease-specific measures of QoL are important as we move forward, but none, including the CHO-KLAT, has been fully tested for use in China. [6] Because of the relatively rare nature of hemophilia, multi-site international trials are becoming increasingly common. Completion of this adaptation will facilitate the inclusion of Chinese children in international studies, in addition to measuring the outcome of factor replacement therapy, during this period of change.

A collaboration between Chinese and Canadian clinicians and researchers began in 2007 with the goal of supporting clinical and research programs in China. The collaborative QoL research group began in China in 2008. [7] The focus of this group was to prepare for clinical research in China, and specifically to focus on cross-culturally adapting outcome measures for the clinical context of China. The purpose of this collaborative study was to complete the adaptation of the CHO-KLAT for use in both rural and urban China. The distinction between rural and urban contexts within China is important due to differences in access to treatment, both in terms of access to expert hemophilia professionals and to availability of factor replacement therapy. Rural was defined as living more than 50 km outside the city where they received their treatment.

## Methods

The development a new language version of an existing measure has been well documented in the literature. [8-11] In China there were 56 different ethnic groups speaking many different dialects to consider. Because there has been 20 years of standardized education in China, and Simplified Chinese is the common written language used for education, there was consensus that a Simplified Chinese version would be accessible to the boys across China. The cross-cultural adaptation process typically involves 5 steps: initial translation (forward) by clinicians whose primary language was Chinese; back translation by a professional translation company; expert committee review and adjudication; field testing (also known as cognitive debriefing); and finally a consensus meeting to approve the initial version of the measure.

### Step 1: Forward translation

The initial translation of the CHO-KLAT into Simplified Chinese was performed by a Chinese physician and a Chinese research assistant, both of whom were working at the Hospital for Sick Children, in Canada. The original CHO-KLAT has been recently revised, and this revised version (denoted as CHO-KLAT_2.0_) was the source used for the translation in this study. The Chinese translation was subsequently reviewed by a pair of clinicians from China.

### Step 2: Back translation

The back translation was prepared by a professional translation company in Toronto.

### Step 3: Expert review and adjudication

The back translation results were compared to the original English version of the CHO-KLAT_2.0_ by the combined China-Canada research team. Discrepancies were reviewed by the local team in China in conjunction with members of the original Canadian CHO-KLAT development team during a face-to-face meeting. At that time it became apparent that there may be other issues relevant to boys in China that were not included in the original English version of the CHO-KLAT_2.0_.

### Modification of the process for China: focus groups to identify additional items

Because the culture and health care systems are vastly different between China and Canada (the original source of the CHO-KLAT_2.0_), it was important to carefully explore the need for potential new items. This was achieved by holding a series of focus groups with boys who had hemophilia and separate focus groups with their parents. We applied the same focus group approach that had been used in the initial development of the CHO-KLAT in Canada. [4] Focus groups were held in Beijing and Chengdu. The latter site was important to include rural children. Each focus group began with a series of open-ended probes, designed to promote discussion of concepts that were important to the quality of life of boys with hemophilia in China. These concepts were recorded and displayed for the group to review. Participants also reviewed all of the CHO-KLAT_2.0_ items and added those that were relevant to them, to the list of concepts on display. The focus group participants later ranked the importance of the concepts. These concepts and rankings were reviewed by the expert committee as part of the review and adjudication process.

The China-Canada research team met again to review the focus group results, and came to consensus on developing an initial version of the Chinese CHO-KLAT_2.0_. The Chinese team included: a pediatric hematologist (RW), a psychiatrist (JZ), 2 hemophilia nurse specialists (XW and YZ), and 2 medical students who assisted with translation (LT and YY). The Canadian team included: a pediatric hematologist (KHL), an experienced educator fluent in both English and Chinese (SML), a clinician-scientist with expertise in the development and testing of outcome measures for use by children (NY), and a research coordinator (TB). The combined China-Canada research team met over a 3-day period to achieve consensus on the item content and develop an initial version of the CHO-KLAT_2.0_ for use in China. Careful attention was paid to the linguistic nuances in the item development. This resulted in a Chinese version of the CHO-KLAT_2.0_ to enter into the cognitive debriefing process.

### Step 4: Cognitive debriefing

The next step in the process, cognitive debriefing, was important to ensure that the Simplified Chinese translation was understood by both boys with hemophilia and their parents in a way that was consistent across respondents, as well as being consistent with the original intent of the items. Cognitive debriefing is an iterative process in which a questionnaire is administered to participants using an interview approach based on Jobe’s framework for assessing cognitive and social-motivational processes. [12] This process was part of the initial CHO-KLAT_2.0_ development. [4] We estimated that approximately 10 boys and their parents would be required for the cognitive debriefing process. The inclusion criteria were: able to read Simplified Chinese, verbally fluent in Chinese, and be between the ages of 7.0 and 17.99 years of age. A mix of boys from both rural and urban regions was recruited from the Beijing Children’s Hospital (BCH). All participants provided written informed consent and attended an interview at the treatment center.

A series of cognitive debriefings were held at BCH. The interview participants were asked to verbalize their thinking around each item and response set as they proceeded through the questionnaire. They were also asked to explain what they thought the items meant. The interviewers kept detailed notes on the participants’ discourses related to each item. The China-Canada research team coded responses to identify word problems, general concept problems or response option problems. The results were reviewed by this team who made decisions regarding modification to individual items after each series of 2 to 3 pairs of debriefings, if necessary. The revisions were evaluated during subsequent repetitions of the process with new participants, until a stable and well understood version of all items was achieved.

### Step 5: Final adjudication

A final adjudication meeting was held to review the results of the cognitive debriefing process and come to consensus on the final version of the CHO-KLAT_2.0_ for China.

### Ethics approval

Ethical approval for this project was received from Beijing Children’s Hospital and Chengdu Women and Children’s Central Hospital. Participants were compensated for their travel.

## Results & discussion

### Steps 1, 2 and 3: Forward and back translations and comparisons

The forward translation was completed in 2008. Two Chinese clinicians (RW and JZ) completed the back translation in 2009. The initial back translation of the Chinese CHO-KLAT_2.0_ was slightly discrepant from the original English version on 12 items. The initial adjudication of discrepancies was completed in August of 2009 and confirmed in mid-October of 2010.

### Modification of the process for China

A series of 3 focus groups were held with boys from both urban and rural China, and an additional 3 groups were held for their parents. These focus groups were led by a psychiatrist at Beijing Children’s Hospital (JZ) whose first language was Chinese and who was experienced in the management of persons with hemophilia in China. Details are presented in Table 1.

**Table 1:** Focus Group Sample Participants

		**First Group**	**Second Group**	**Third Group**	**Total**
**Date**		August 2009	October 2009	October 2010	
**Region**		Urban	Rural	Rural	
**Boys**	**Sample**	8	4	8	20
**Boys**	**Age range**	7-16 years	6-11 years	5-13 years	5-16 years
	**(mean)**	(10.75)	(9.12)	(9.38)	(9.88)
**Boys**	**Education**	0 pre-school	0 pre-school	1 pre-school	1 pre-school
		4 primary	4 primary	6 primary	14 primary
		3 middle	0 middle	1 middle	4 middle
		1 senior-middle	0 senior-middle	0 senior-middle	1 senior-middle
**Boys**	**Severity**	5 moderate	2 moderate	4 moderate	11 moderate
		3 severe	2 severe	4 severe	9 severe
**Parents**	**Sample**	10	13	8	31
**Total**		18	17	16	51

A total of 20 boys and 31 parents were involved in these focus groups. The boys ranged in age from 4.5 to 16 (mean 9.4) years. Nine had severe (defined as <1% clotting factor) and 11 had moderate hemophilia (defined as 1 to 5% clotting factor). Twelve of the 20 were from rural areas. All items were endorsed by at least 2 focus groups and 77% of the CHO-KLAT_2.0_ items were endorsed by at least half of the focus group. These groups confirmed that all 35 CHO-KLAT_2.0_ items were relevant in both rural and urban China. They also identified 47 potential additional items. These were aggregated into 9 concepts during the expert panel meeting in Beijing (October 22^nd^, 2010) and developed into 9 new items. These were common to many focus groups. Because most of these were conceptually distinct from the other CHO-KLAT_2.0_ items they were combined in an add-on module. These items primarily focused on the socio-economic impact of hemophilia and its treatment, thus the module was named the Socio-Economic Context (SEC) module. This module included items about stigma (e.g., *I felt that other people did not treat me as well as they treated other children*), opportunities for stable employment (e.g., *I worried that I will not be able to find a good job when I grow up*), and access to treatment due to cost (e.g., *I worried about the high cost of factor for my hemophilia treatment*).

### Step 4: Cognitive debriefing

Seven child-parent pairs were debriefed between October 24^th^ and 26^th^ 2010, by the combined Chinese and Canadian teams. All of the boys had hemophilia A, 2 had moderate and 5 had severe hemophilia. Six boys had experience with low-dose prophylaxis (10 units/kg twice per week) for 3 months (short-term) at the time of the interviews. The group of caregivers consisted of 6 mothers, and 1 father. Three items were not consistently interpreted by the first 2 child-parent pairs, thus modifications were made to these items after the second pair was completed. Two of these continued to be problematic and were revised a second time after the 5^th^ debriefing pair. Six additional items were also refined at that time. After the 7^th^ debriefing pair, minor modifications were made to 2 items to improve the precision of the wording. The response set was well understood by all participants.

Six additional child-parent pairs were debriefed between November 2010 and June 2011, by the Chinese team independently. The group consisted of 5 boys with hemophilia A and 1 with hemophilia B. In this group, 4 had moderate and 2 had severe hemophilia. Four boys had experience with low-dose prophylaxis (10 units/kg twice per week) for 3 months (short-term) at the time of the interviews. The group of caregivers consisted of 4 mothers and 2 fathers. Final linguistic improvements were made after the 13^th^ cognitive debriefing pairs.

The total cognitive debriefing sample ranged in age from 8 to 17.5 years (mean age of 12.5 years) and 6 of the 13 lived in rural areas. Seven of the boys had severe hemophilia and 6 had moderate hemophilia.

### Step 5: Final adjudication

The combined results of the 13 cognitive debriefing pairs were reviewed at 2 adjudication meetings at BCH on June 13^th^ 2011 and October 22^nd^, 2011. Minor concerns with wording were identified with 5 CHO-KLAT_2.0_ items; 3 of these had been revised at least once previously. In addition, minor concerns were identified with 2 SEC items. Thus subtle refinements in the linguistic nuances of 7 items were made at this time. Based on expert consensus, the Chinese version of the CHO-KLAT_2.0_ and associated SEC module was finalized. Throughout the process a total of 18 items underwent some modification to produce a final version that could be consistently understood by boys in China.

## Conclusion

This paper presents the process of adapting a quality of life measure for use in China. During the process we identified the need to return to item generation to determine if additional items were necessary. This process resulted in a new Socio-Economic Context module with 9 important items that are unique to the context of current hemophilia care in China. Although this process was conducted in China, the lessons learned are likely to be transportable to other developing countries. It is important to note that, in order to achieve the goals of the initiative, there needed to be significant time investments made by a diverse group of experts from both China and Canada. The strength of the China-Canada collaborative relationship was essential to the success of this project.

Through the process we confirmed that all 35 CHO-KLAT_2.0_ items were relevant in China. The nuances of the language proved challenging, and required more cognitive debriefings than initially expected. However, diligence and persistence resulted in a stable version of the Chinese CHO-KLAT_2.0_ that was confirmed by consensus. The content validity of the CHO-KLAT_2.0_ has been confirmed.

The Chinese version of the CHO-KLAT_2.0_ requires validation, and once this has been completed, the measure will enable comparison of the QoL of Chinese boys with hemophilia to those in other countries. It will also be valuable for tracking the changes in QoL over time as more treatment becomes available in China. We expect that the SEC module will be particularly valuable to track changes over time within China.

In conclusion, the final product of this study is a Chinese version of the CHO-KLAT_2.0_ that is ready to enter validity testing. Construct validity of this measure will be conducted as part of a future study.

## Abbreviations

CHO-KLAT: Canadian Hemophilia Outcomes – Kids Life Assessment Tool; QoL: Quality of Life; SEC: Socio-Economic Context; WFH: World Federation of Hemophilia.

## Competing interests

This group has no competing interests to declare.

## Authors contributions

RW, KHL, TB, MCP, JS, VB and NLY participated in the design of the study. JZ, RW, XW, XL, MZ, KHL, TB and NLY were integral to the data collection. LT and MH assisted with linguistic translation during key consensus meetings. NLY conducted the analysis in collaboration with RW. RW, JZ, KHL, TB, JS and NLY participated in the key consensus meetings. All authors read and approved the final manuscript.

## Authors information

This paper was possible due to the combined efforts of hematologists (RW, JS, XL and MZ) and psychiatrics (JZ) practicing in China, Canadian hematologists (KHL, MCP and VB), a clinical epidemiologist (NY), a senior research coordinator (TB) and medical trainees who supported the initiative (LT and MH). This work sought to represent the perspectives of patients and families.
